# Clinical Application of Infrared Thermography in Rheumatic Diseases: A Systematic Review

**DOI:** 10.31138/mjr.271024.ita

**Published:** 2025-01-30

**Authors:** Naveenkumar Nallathambi, Rahul Bisaralli, Shriganesh Palanikumar Naidu, M S Mallikarjunaswamy, P Praveen, Mahabaleshwar Mamadapur

**Affiliations:** 1 Institute of Internal Medicine, Madras Medical College, Chennai, Tamilnadu, India; 2 Department of Clinical Immunology and Rheumatology, SDM College of Medical Sciences and Hospital, Dharwad, Karnataka, India; 3 Department of Electronics and Instrumentation Engineering, Sri Jayachamarajendra College of Engineering, JSS Science and Technology University, Mysuru, India

**Keywords:** infrared thermography, rheumatic diseases, diagnosis, monitoring, treatment

## Abstract

**Aim:**

This systematic review aims to evaluate the clinical applications of infrared thermography (IRT) in rheumatic diseases (RDs), focusing on its potential as a non-invasive, cost-effective, and reliable tool for diagnosis, monitoring, and treatment to improve patient outcomes.

**Methods:**

A systematic literature review was conducted following the PRISMA guidelines. A comprehensive search strategy was implemented using various databases, namely Medline/PubMed, Scopus, Web of Science, Google Scholar, PubMed Central, Cochrane Library, and ScienceDirect. After screening, data extraction and quality assessment were performed to synthesise the findings and evaluate the methodological quality of the included studies.

**Results:**

The systematic review included 51 studies comprising 7 randomised controlled trials and 44 observational studies. IRT demonstrated utility in various RDs. In osteoarthritis, it detected elevated temperatures in affected joints, correlating with pain intensity. For rheumatoid arthritis, IRT was effective in diagnosing active synovitis and monitoring disease progression, although its effectiveness was limited in small joint assessments. In Sjögren’s syndrome, IRT differentiated dry eye aetiologies, while in fibromyalgia, the studies yielded mixed results. IRT effectively assessed arthritis in juvenile idiopathic arthritis and aided in detecting disease activity, monitoring progression, and evaluating treatment responses in scleroderma and Raynaud’s phenomenon. Additionally, IRT showed potential in assessing therapeutic interventions across several conditions.

**Conclusion:**

IRT showed significant potential as a non-invasive tool for diagnosing, monitoring, and evaluating treatment in RDs. While its effectiveness varied by condition, IRT complemented existing methods. Further research is needed to standardise protocols and confirm its clinical utility.

## INTRODUCTION

Rheumatic diseases (RD), characterised by chronic joint and tissue inflammation, affect about one-third of the global population. In Europe alone, they impose a financial burden of nearly 200 billion euros annually. The World Health Organisation (WHO) cites these diseases as the second leading cause of disability worldwide.^1^ RD, affecting 9.8% to 33.2% of the population, lead to 15–45% of primary care visits. They have been identified as a major cause of long-term work absences, accounting for over half of sickness cases. Recognised by the United Nations and WHO, initiatives like the Bone and Joint Decade 2000–2010 aimed to improve awareness and management.^2,3,4^

Early detection and monitoring are essential for the effective management of rheumatic diseases (RD). Infrared thermography (IRT) has evolved over the past thirty years and has proven effective in diagnosing and monitoring joint diseases, offering greater sensitivity and reliability in detecting inflammatory processes.^5–11^ IRT is a non-destructive testing technique that uses infrared radiation (700 nm to 1 mm) to detect over-heating in electrical circuits and pipes. By visualising surface temperature distributions, IRT enables non-intrusive evaluations. Thermographic cameras capture and convert thermal radiation into visual images in the mid-infrared range (0.9 to 14 micrometres).^12,13^ Despite its ability to detect various bodily dysfunctions through core temperature measurement, IRT lacks sufficient scientific evidence for its effectiveness in diagnosing specific musculoskeletal diseases by evaluating skin surface temperature. While IRT shows promise in identifying inflamed or dysfunctional joints and detecting dysfunctional joint patterns, further research is needed to connect these patterns with painful symptoms. Establishing this link could enable healthcare professionals to develop therapeutic strategies that address both neuropathic and nociceptive aspects of chronic musculoskeletal pain, potentially leading to improved treatment outcomes.^14,15^

This systematic review aims to comprehensively evaluate the clinical applications of IRT in diagnosing, monitoring, and treating various rheumatic diseases. It seeks to assess the potential of IRT as a non-invasive, cost-effective, and reliable tool in rheumatology. By compiling existing evidence, the review aims to enhance early detection and improve disease management, ultimately leading to better patient outcomes.

## METHODS

The systematic literature review was conducted following the guidelines set by the preferred reporting items for systematic reviews and meta-analyses (PRISMA).^16^

### Inclusion and exclusion criteria

To include the maximum number of publications, no time limit was applied to the studies considered. Articles were deemed eligible if they demonstrated a clinical application of IRT in rheumatic diseases at any level of clinical evidence. Additionally, they had to be written in English and published with IRT used for diagnosing rheumatic diseases, monitoring disease progression, or evaluating response to treatment. Exclusion criteria considered were articles written in languages other than English, studies conducted on animals, preclinical studies, reviews, case reports, retracted articles, book chapters, clinical applications related to other diseases or conditions, protocols, abstract papers, theses, and studies with inadequate information in the methodology section and study subjects.

### Search strategy

A systematic literature search was conducted using search terms such as “Infrared thermography AND Rheumatic diseases,” “Thermography AND Rheumatic diseases,” “Infrared thermography AND Rheumatoid arthritis,” and “Infrared Camera or Thermal Camera AND Rheumatic diseases,” along with relevant variations (**Supplementary Table 1**). The initial searches were performed on primary databases including Medline/PubMed, Scopus, and Web of Science to ensure a comprehensive exploration of the topic. Additionally, supplementary sources such as Google Scholar, PubMed Central, Cochrane Library, and ScienceDirect were consulted to validate findings and identify any overlooked relevant studies. Furthermore, select review articles pertaining to the subject were cross-referenced to procure additional references.

### Study screening

Following the search, two reviewers independently evaluated titles and abstracts to remove papers not meeting the inclusion criteria. Duplicate entries were removed across databases, and full-text articles meeting the criteria were obtained.

### Data extraction and synthesis

Two impartial assessors extracted necessary information from the studies included. Each study was thoroughly read, and critically evaluated, and data were collected. The collected data included general information about each study (such as the author and publication year), study type, participant demographics, sample size, type of infrared thermographic camera, thermography protocol, study findings related to thermography, and the clinical application of thermography. A comprehensive synthesis of the collected data was conducted, summarising the findings and emphasising the potential clinical uses of IRT in managing rheumatic diseases.

### Quality assessment

The methodological quality of the included studies was assessed using standardised tools appropriate for each study design. For cohort and case-control studies, Newcastle-Ottawa Scale (NOS) was used, which evaluates studies on three domains namely selection, comparability, and outcome (for cohort studies) or exposure (for case-control studies). The NOS uses a star system, with a maximum of 9 stars indicating the highest quality.^17,18^ Cross-sectional studies were evaluated using an adapted version of the NOS, with a maximum score of 10 stars across the selection, comparability, and outcome domains.^19^ Diagnostic studies were assessed using the Quality Assessment of Diagnostic Accuracy Studies-2 (QUADAS-2) tool, which evaluates the risk of bias and applicability concerns in four key domains: patient selection, index test, reference standard, and flow and timing.^20^

For randomised controlled trials, the Jadad scale was employed, which assesses randomisation, blinding, and withdrawals/dropouts, with scores ranging from 0 to 5. Higher scores indicate better quality.^21,22^ The single case series identified was evaluated using the Joanna Briggs Institute (JBI) Critical Appraisal Checklist for Case Series, which includes 10 criteria addressing various aspects of study design and reporting.^23^ Reviewers conducted the quality assessments independently, resolving discrepancies through discussion or, if necessary, consultation with a third reviewer. The results of these assessments were used to inform the interpretation of study findings and assess the overall strength of evidence in the present systematic review. Due to data inconsistencies, a meta-analysis of the results was not conducted.

## RESULTS

### Study selection and characteristics

**Figure 1.**
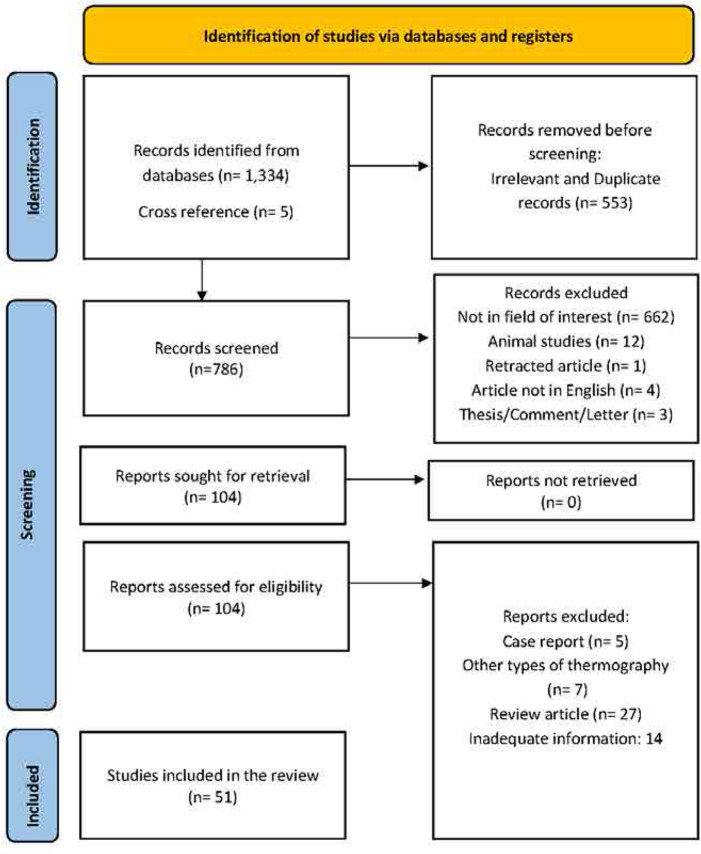
PRISMA chart summarising the selection of studies.

An initial literature search yielded a total of 1,334 studies, along with 5 additional articles identified through cross-referencing. After removing 553 duplicates, 786 studies remained. Subsequently, 682 studies were excluded after a review of the abstracts. A thorough evaluation of the full texts resulted in 104 studies being considered for eligibility. Further exclusions included 5 case reports, 7 studies using other types of thermography, 27 review articles, and 14 studies with inadequate information. Ultimately, 51 studies met all criteria, comprising 7 randomised controlled trials and 44 observational studies. The progression of study selection, from the initial search to the final studies included in the data synthesis, is depicted in a PRISMA flow diagram (**Figure 1**). These studies collectively involved 2,463 patients, with a mean age ranging from 1.5 to 79 years and an increased female predominance.

### Quality assessment of selected studies

The systematic review included a mix of cohort, case-control, cross-sectional, and diagnostic studies examining the clinical application of IRT in RD. The quality assessment of included studies revealed generally moderate to high quality across different study designs. For cohort studies, scores ranged from 5 to 9 out of 9 possible points, with most studies scoring 7 or higher. Case-control studies showed similar quality, with scores between 6 and 9 out of 9. Cross-sectional studies demonstrated more variability, with scores ranging from 5 to 10 out of 10 with most studies achieving good quality scores. The QUADAS-2 assessment for diagnostic studies indicated mixed results, with some studies showing a low risk of bias and high applicability, while others had unclear or high risk in certain domains. Randomised studies evaluated by the Jadad score ranged from 2 to 5 out of 5, suggesting moderate to high quality for most included randomised trials. The single case series evaluated using the JBI Critical Appraisal Checklist showed partial to full adherence to most criteria, indicating reasonable quality for this study type. Overall, while there was some variability in study quality, most included studies demonstrated sound methodological approaches, supporting the reliability of findings regarding the application of IRT in RD (**Supplementary Table 2–7**).

The review covers three main areas of application namely diagnostic, monitoring and evaluation, and therapeutic. The basic characteristics of the reviewed studies and key thermography findings for each condition are summarised in **Table 1** and **Table 2**, and **Supplementary Table 8** and **Supplementary Table 9**, respectively.

**Table 1.** Basic characteristics of the included studies (different types of arthritis).

**Author/year**	**Study design**	**Study group**	**No of patients**	**Age (Year) (Mean range)**	**F %**	**Disease/conditions**	**Thermography protocol and infrared camera model**	**Study findings**
Alfieri et al., 2020^25^	Case series	Individuals with unilateral knee OA	11	63.1	NR	Knee OA	Participants exposed their lower limbs and acclimated for 20 minutes at 21.3°C and 63.4% humidity. Thermal images were taken with a FLIR T650SC camera from 2 meters away, assuming skin emissivity of 0.98. FLIR Tools software measured average temperatures in thigh, leg, and knee ROIs.	There was a significant temperature difference between affected and unaffected knees in the knee ROI (0.3°C higher in affected knee.
Arfaoui et al., 2012^26^	Obs case-control study	Patient with knee OA	10	21.50	0	Knee OA	Thermal images of both knees were captured with an infrared camera (e.g., CEDIP TITANIUM HD560M ) before and after a 5-minute treadmill run at 8 km/h, and average temperatures at defined knee regions were measured from the images.	IRT showed asymmetrical thermal patterns between affected and unaffected knees in OA patients. After exercise, knee temperature increased linearly for 5 minutes, then plateaued. Affected knees were about 2°C warmer at rest and higher temperatures correlated with greater pain intensity.
		Healthy controls	12	23.08		NA		
Balay-Dustrude et al., 2023^65^	PCS	Asx exercise cohort	26	11.5	69.2	NA	Thermal images of lower limbs were taken using Fluke™ and FLIR™ cameras. Participants rested for 10 minutes at 22.2°C. Asymptomatic subjects were imaged at baseline, post-walking, and post-resting, while symptomatic knee pain subjects were imaged with both cameras.	In the asymptomatic exercise cohort, post-physical activity thermal imaging showed significant temperature changes that did not consistently return to pre-activity levels after rest. In the symptomatic knee pain cohort, thermal cameras accurately detected joint inflammation and assessed joint temperature with high sensitivity (80%) and specificity (84.2%) in the anterior knee view.
		Sx knee pain cohort	43	11.2	62.8	Sx knee pain		
Brito et al., 2020^52^	QE	OA group (Men)	7	75.0	NA	OA	IRT measures skin temperature before and after an exercise session. Thermograms of the anterior and posterior views of the lower body were taken, analysing six regions of interest: knee, anterior/posterior thigh, anterior/posterior leg, and popliteal region. Subjects, wearing shorts, were imaged from 3-4 meters away using a FLIR T335 thermal camera under ambient conditions of 28.2 ± 0.5°C and 48.1 ± 1.2% humidity.	Subjects with OA had higher pre-exercise skin temperature in the thigh compared to controls. Post-exercise, their thigh temperature decreased. Conversely, pre-exercise knee temperature was lower in osteoarthritis subjects but increased post-exercise.
Bhowmik et al., 2016^27^	Obs	Patients with knee OA	15	55.1	73.3	OA	A high-resolution FLIR T650sc thermal camera was used, placed within 2 meters on a tripod, and aligned at 90° to the knee. Environmental conditions were 22–25°C and 40–60% humidity. Patients stabilised for 15 minutes and avoided stimulants and intense activities for 24 hours prior.	In OA unilateral joint involvement results in higher inflammation spread and elevated temperature, standard deviation, and pixel intensity contrast in the affected knee. Conversely, RA affects bilateral joints, displaying uniform inflammation spread and comparable temperature and pixel intensity distributions in both knees.
		Patients with knee RA	15	49.4	100	RA		
De et al., 2023^28^	CSS	Patients with Sx knee OA	60	61.4	36.7	OA	A FLIR T1020 IR camera captured images 1 meter perpendicular to the knee at baseline (T0), immediately after a 2-minute knee flexion-extension exercise (T1), and 5 minutes post-exercise (T2). ResearchIR software analysed temperatures of the entire knee and five specific ROIs.	Temperature decreased immediately after exercise, from 32.13°C at T0 to 31.86°C at T1, and remained lower than baseline at T2 (31.94°C). Different ROIs exhibited varying temperature trends. Patients with poor clinical status showed a lower temperature response, and women exhibited a greater temperature decrease than men.
Denoble et al., 2010^49^	CSS	Patients with knee OA	15	61.9	100	OA	Infrared imaging of the knee was performed with a Meditherm Med2000™ Pro camera. Consistency was assessed by calculating ICCs from two images taken six months apart in Controls. Average temperatures for five knee regions were determined using WinTes software.	The knee infrared thermal imaging procedure demonstrated long-term reproducibility with high ICCs for the various regions of interest in Controls. Cutaneous temperature of the patella (knee cap) yielded a significant correlation with severity of knee rOA.
Devereaux et al., 1985^60^	PCC	Patients with RA	20	53.1	70	RA	In a controlled environment (20 ± 0.5°C, 50 ± 10% humidity), subjects rested for 15 minutes before infrared imaging with an AGA 680M system. The camera, 1 meter away, captured images of wrists, knees, elbows, and ankles.	Thermography showed a significant correlation with the Ritchie articular index, Mallya score, grip strength, morning stiffness, erythrocyte sedimentation rate, and pain score.
Frize et al., 2011^32^	CSS	Patients with RA	13	19–70	69	RA	After a 15-minute acclimation, images were captured at 30 fps using a solid-state microbolometer (320×420 pixels, 7.5–13μm range) with a 24mm germanium lens and 24°×18° field of view, focusing on hands, arms, knees, and feet.	RA patients showed significantly higher temperatures in all hand joints and parts compared to controls. The best joints for RA detection were the 2nd and 3rd MCP joints and knees.
		Controls	18		44	NA		
Gatt et al., 2019^33^	Case-control study	Patients with RA	31	60.19	93.5	RA	Images were taken with a Flir T630 camera, following American Thermology Association guidelines. Participants acclimatised to 23°C for 20 minutes and extended their hands. Images of palms and dorsal hands were captured from 1.5 meters and analysed with FLIR software.	RA patients without active synovitis had significantly higher mean temperatures in the palms (31.4°C) and fingers (30.22°C) compared to healthy controls (palms 29.37°C, fingers 27.16°C). Logistic regression models indicated that increased temperatures in these areas were significant predictors of RA.
		Healthy controls	51	36	76.5	NA		
Gatt et al., 2020^34^	CSS	RA patients in remission	32	60.19	90.6	RA	Participants acclimated supine for 15 minutes. Thermal images of the plantar feet were captured from 1.5 meters with a FLIR T630 camera, and mean temperatures were analysed using FLIR ResearchIR Max software.	RA patients in clinical and radiological remission showed significantly higher mean temperatures in all forefoot and heel regions than healthy controls.
		Healthy controls	51	36	76.5	NA		
Gizińska et al., 2021^35^	CSS	Patients with RA	81	57.4	NR	RA	Surface temperature of the feet was measured with a ThermaCAM SC640 camera at 21°C and 40±10% humidity. Emissivity was set to 0.98, with calibration 20 minutes prior. The camera, positioned 50 cm above, captured images of seven ROIs on the dorsal.	Significant differences in mean temperatures were found between the study group and the control group.
Goldie et al., 1974^63^	RCT	Patients with RA	24	40–79	83.3	RA	An AGA Thermovision Model 680 thermograph recorded heat from the back of both hands, with a reference temperature of 32°C. Patients equilibrated for 15–20 minutes in a room with minimal people and no meals or smoking allowed beforehand.	Thermographic findings corresponded well with the subjective evaluation of naproxen effectiveness, with 13 patients showing thermographic improvement compared to 6 with the placebo.
Hegedus et al., 2009^54^	RCT	Active LLLT group	18	49.48	81.5	Patients with knee OA	In a room at 21–23°C and 70–80% humidity, patients rested for 15 minutes. The Medial and lateral readings were taken weekly after the second treatment, and at 2 weeks and 2 months using an AGA infrared camera.	Weekly thermograms showed increasing temperatures and warmer areas in the active LLLT group, but not in the placebo group. At the 2-month follow-up, temperatures remained elevated by at least 0.5°C in patients with pain relief, including on the non-treated control side.
		Placebo LLLT group	9					
Jones et al., 2018^61^	CSS	Patients with RA	49	57.9	79.6	RA	A FLIR T300 camera measured temperatures over MCP and PIP joints to determine mean absT. In a 22 ± 0.5°C room, patients rested for 15 minutes with the camera fixed at 0.5 meters and a standardised hand splint. Results were quantified as absT.	Thermographic analysis showed no correlation with clinical measures of disease activity. In patients with RA, no statistically significant relationship was found between joint temperature and clinical assessments of disease activity.
		Healthy controls	30	26.3	66.7	NA		
Lasanen et al., 2015^42^	CSS	Children with symptoms of mono- or polyarthritis*	58	1.5–17.0	NA	JIA, SLE, or joint pain symptoms	A FLIR A325 infrared camera (320 x 240 pixels, 0.05°C resolution) was used, calibrated with a skin emissivity of 0.98. Patients stabilised for 15 minutes in a 21°C room. The camera imaged knees and ankles from 1 meter, analysing ROIs for mean and maximum temperatures, SD, and HDI.	In inflamed ankle joints, both maximum and mean temperatures were significantly higher compared to non-inflamed ankles.
Lerkvaleekul et al., 2017^40^	CSS	JIA patients with active arthritis	30	10.2	NR	JIA	IRT was performed in a draft-free room at 22 ± 0.5°C and 50 ± 10% humidity using a FLIR E60 camera with 0.045°C sensitivity. Patients removed wrist clothing and bracelets 15 minutes prior. The camera, positioned 1 meter from the wrist, had a skin emissivity of 0.98. The ROI centred on the 3rd metacarpal, bounded by the ulna and radius.	The arthritis group showed higher Tmean and Tmax skin surface temperatures in the ROI than the inactive and healthy groups. These temperatures were particularly elevated in the moderate to severe arthritis group. The HDI was also higher in this group compared to healthy controls.
		JIA patients with inactive disease	16	7.7		JIA		
		Healthy controls	15	9.0		NA		
Lindberg et al., 2021^48^	Obs	Patients suspected of having RP	41	56.2	7.3	RP	Participants immersed their hands in 10°C water for 60 seconds. Rewarming was recorded with a FLIR SC600 camera and analysed for baseline, post-cooling, halfway rewarming, end temperatures, time to end temperature, and recovery percentage.	The tested algorithm's performance was noninferior to the FSP test with a 0.05 cut-off, showing 69% sensitivity, 58% specificity, and 66% accuracy. The FSP test had 77% sensitivity, 37% specificity, and 59% accuracy.
Nwaizu et al., 2020^41^	CSS	Children JIA	20	11.2	55	JIA	Thermal imaging was performed in a room at 23.6°C and 33% humidity. After 10 minutes of acclimatisation, knee images were captured with a FLIR T630sc camera from 1 meter, recording 600 images over 20 seconds at 30 fps.	The median temperature of knees with active inflammation was significantly higher than that of inactive knees. In 6 of the 8 participants with one actively inflamed knee, thermal imaging accurately identified the warmer knee. Additionally, in 16 of the 20 participants, thermal imaging detected the warmer knee compared to their respective reference regions.
Morales-Ivorra et al., 2022^37^	CSS	Patients with RA	146	57	80.1	RA	Thermal images of the hands were captured with a Flir One Pro or Thermal Expert TE-Q1 camera with a 6.8mm lens during outpatient visits before ultrasound and physical exams. The dorsal aspect of both hands, with fingers spread, was recorded without a standardised distance.	ThermoJIS moderately correlated with ultrasound scores for synovial hypertrophy and power Doppler. It achieved an AUROC of 0.78 for detecting active synovitis and showed significantly higher values in rheumatoid arthritis patients with ultrasound-detected active synovitis during clinical remission.
De Marziani et al., 2023^53^	CSS	Patients symptomatic knee OA	40	61.3	35	OA	A FLIR T1020 thermographic camera, positioned 1 meter from the knee, captured anterior images focusing on five ROIs—medial, lateral, medial patella, lateral patella, and suprapatellar— allowing for the extraction of mean temperatures for both the entire knee and each ROI.	Higher knee temperatures were linked to younger age, male sex, higher BMI, and worse objective knee scores, while lower temperatures were observed in patients with neuropathic pain. The patellar area consistently remained colder than other knee regions.
Pauk et al., 2019^55^	Obs	Patients with RA	30	56.5	NR	RA	Subjects acclimatised for 15 minutes at 23 ± 1°C and 55% humidity. Baseline hand temperature was recorded twice for 30 seconds with a FLIR E60bx camera. After 30 seconds in 0°C water, rewarming was monitored for 180 seconds. Two video recordings of dorsal fingers were taken per patient	Baseline finger temperature was similar between RA patients and healthy participants (p > 0.05). The cold provocation test increased Tmax-Tmin contrast, showing significant differences in the thumb, index, middle, and ring fingers post-cooling and rewarming (p < 0.05). The index finger had the highest ROC value (0.68) for distinguishing RA patients based on Tmax-Tmin post-cooling.
		Healthy participants	22	49.8	NR	NA		
Pauk J et al., 2019^56^	RS	Patients with high DA	50	51.9	83.3	RA	Thermography with a FLIR E60bx camera (320 × 240 pixels, sensitivity <0.405 °C) was performed after 15 minutes of acclimation in a 23 °C, 55% humidity room. The camera, positioned 1 m from the fingers, captured static, cooling (0 °C for 5 seconds), and rewarming (180 seconds) images, following the Glamorgan protocol.	In static measurements, the average finger temperature of RA patients with moderate and high DA was similar. Post-cooling, fingers of moderate activity patients cooled more. Post-rewarming, high DA patients showed slower heating, indicating impaired vascular flow. The ΔTR and SR were significantly smaller in high DA patients. ΔTR negatively correlated with DAS28 and tender joints in moderate DA patients, while post-cooling temperatures in high DA patients correlated with rheumatoid factor and anti-CCP antibodies
		Patients with moderate DA	16	55.4				
		Healthy controls	42	54.1	NR	NA		
Snekhalatha et al., 2015^36^	CSS	Patients with RA	30	45.3	75	RA	Participants acclimatised their hands for 15 minutes in a 20°C room with 45–50% humidity. Thermal images of both dorsal and ventral views were captured with a ThermaCAM-T400 camera from 1 meter, with a range of −20°C to 1200°C and sensitivity of 0.05°C.	RA patients demonstrated significantly higher HDI and TI values compared to healthy controls. The mean skin temperature at the MCP3 joint showed strong correlations with the majority of the extracted hand features. Extracted statistical features from thermal images exhibited markedly elevated values in RA patients relative to controls.
		Healthy subjects	15	45.5	75	NA		
Tan et al., 2020^57^	CSS	Patients with RA	37	56.5	76	RA	The thermography procedure was conducted at 22°C in a draft-free environment. A FLIR T640 camera, positioned 50 cm above the hands, captured dorsal views. Joint areas were manually selected, and temperature measurements were taken.	Joints exhibiting ultrasound-detected inflammation showed markedly elevated temperature readings across all thermographic parameters. The comparison between inflamed and non-inflamed joints on ultrasound revealed substantial effect sizes (>0.80) for numerous thermographic parameters.
Tan et al., 2024^59^	CSS	Patients with RA	30	57.7	77	RA	Using a FLIR T640 thermal camera with <30 mK sensitivity at 30°C, images were taken 50 cm from the medial, lateral, posterior, and anterior aspects of each elbow. Regions of interest were manually segmented, recording MIN, MAX, and AVG temperatures.	Thermographic parameters (MIN, MAX, AVG) significantly correlated with TPD score at both elbows. These parameters correlated with TGS scores at the right elbow.
Umapathy et al., 2020^38^	Case-control study	Patients with RA	30	46.93	NR	RA	The thermography protocol used a ThermaCAM-T400 (FLIR) to capture posterior-anterior knee images in a room at 20°C and 45–50% humidity. Subjects acclimated for 10 minutes before imaging. Images were taken from 1 meter away with subjects standing and sitting (knees flexed at 90°).	In RA patients, there was a 5.94% increase in average skin temperature compared to normal. Additionally, erythrocyte sedimentation rate and C-reactive protein exhibited a significant positive correlation with thermal imaging parameters.
		Healthy controls	30	45.23	NR	NA		
Varjú et al., 2004^50^	CSS	Patient with nodal hand OA	91	69.5	80	OA	The thermography protocol used a Compix PC2000e in a 21°C draught-free room. Subjects sat with uncovered hands for 15 minutes. Hands were placed on black velvet, with the camera 15 inches above. An 8-second thermal image focused on 8x8 mm regions over the DIP, PIP, and MCP joints of digits 2–5.	The mean joint temperature decreased with increasing rOA severity, as defined by the KL scale. KL0 joints had a significantly different mean temperature compared to all other KL grades. There was a strong association between rOA and joint surface temperature. earliest detectable radiographic disease (KL1) exhibited a higher surface temperature than KL0 joints and higher than any other KL grade.
Vasdev et al., 2023^39^	Case-control study	Patients with RA	50	44.04	80	RA	Using a Testo® 885 infrared camera in a 23°C environment, images were taken from the lateral aspect of knees flexed at 90 degrees from 0.5 meters after 20 minutes of acclimation with the area uncovered. The knee and thigh areas were defined for temperature analysis.	Mean knee temperature and knee-thigh temperature differential were significantly higher in RA cases (+1.08°C) than controls (−0.5°C). PDUS inflammation correlated with higher knee temperatures. A knee-thigh differential of 0.15°C was the optimal cutoff, with 100% sensitivity and 94.35% specificity.
Vinson et al., 2020^62^	CSS	Patients with RA	53	61.6	81		The protocol used a FLIR One® infrared camera (0.1°C sensitivity) and GipsVision software to detect 10 ROIs: wrists, MCP, and PIP joints. ΔT between each joint and the ipsilateral forearm was calculated, and thermal images were analysed for arthritis.	The ΔT-joint was higher in tender, swollen joints and in US syno0vitis detected in B mode. However, the mean ΔT-joint was not associated with the PDUS category. Synovitis identified through thermal image readings was linked with a PDUS grade 3.
Zhang et al., 2023^51^	PS	Patients diagnosed with knee OA	50	62.53	80	Knee OA	The protocol used a FLIR SC620 infrared imager in a 25°C±1°C, 50%±10% humidity environment. After 20 minutes of rest, patients stood while the camera, positioned 72 cm from the knee, captured two images of the anterior knee with a 5-second interval on the initial day and after one month.	Knee temperature was higher in the moderate-severe osteoarthritis group than in the mild group. Knee temperature positively correlated with the degree of cartilage wear observed on ultrasound, as well as with pain scores (VAS) and the WOMAC OA index
Zhao et al., 2022^42^	PCS	JIA group	51	9.0	65	JIA	The protocol used a Fluke TiR32 Thermal Imager to capture knee, ankle, and mid-tibia images from four views (anterior, posterior, medial, lateral) after a 10-minute rest. MATLAB software analysed the images and calculated TAWiC by subtracting mid-tibia temperature from joint temperature.	TAWiC was significantly higher in inflamed joints compared to uninflamed joints.

* Children with symptoms of mono- or polyarthritis, including those with diagnosed JIA, SLE, or joint pain symptoms. ROI: Regions of interest; IRT: Infrared thermography; OA: Osteoarthritis; RA: Rheumatoid arthritis; ICCs: Intraclass correlation coefficients; MCP: Metacarpophalangeal; absT: absolute temperature; HDI; Heat Distribution Index; JIA: juvenile idiopathic arthritis; NR: Not reported; NA: Not available; DA: Disease activity; mK: milli-Kelvin; MIN: Minimum; MAX: Maximum; AVG: Average; DIP: Distal interphalangeal; PIP: Proximal interphalangeal; ΔT: Temperature difference; TAWiC: Temperature after within-limb calibration; SLE: Systemic lupus erythematosus; VAS: Visual Analog Scale; PCC: Prospective: comparative: cohort; RS: Retrospective study; Obs: Observational; PS: Prospective study; PCS: Prospective cohort study; QE: Quasi-experimental; CSS: Cross-sectional study; Sx: Symptomatic; Asx: Asymptomatic.

**Table 2.** Basic characteristics of the included studies (other rheumatic diseases).

**Author/Year**	**Study design**	**Study group**	**No of patients**	**Age (Year, mean/range)**	**F %**	**Disease/conditions**	**Thermography protocol and infrared camera model**	**Study findings**
Abreau et al., 2016^24^	RS	Patients with dry eyes: SS, EDE, ADDE	SS: 10, EDE: 10, ADDE: 10	SS: 51.8, EDE: 48.4 ADDE: 46.1	SS: 100, EDE: 50 ADDE: 60	SS, EDE, ADDE	In a 24°C, 40% humidity chamber, a FLIR A40 camera was positioned 25 cm from the subject's eye. Subjects blinked and held their eyes open for 5 seconds, repeating 4–5 times. Videos were recorded at 30 fps for 25 seconds, capturing ROIs including the central cornea, conjunctiva, upper eyelid, and periorbital skin.	Compared to normal eyes, dry eyes exhibited lower initial central OST and reduced temperatures in the central upper eyelid
		Normal eyes group	10	50.1	40	NA		
Agazzi et al., 2018^43^	Obs	Patients with JLS	47	13.4	63.8	JLS	Patients were examined with an infrared camera (ThermaCAM PM695) in a controlled temperature room after 20 minutes of acclimatisation. Lesions were considered active if they were 0.5°C warmer than the surrounding area or contralateral limb.	IRT detected a decrease in hyperthermia. The improvement detected by IRT over time was comparable to the changes in mLoSSI scores.
Bali et al., 2011^69^	RCT	Iloprost group	10	57.8	70	RP and SSc	Digital temperature was measured with a FLIR Thermacam PM595 before and after infusions. After 15 minutes acclimation (22–24°C, 40–50% humidity), temperatures at proximal and distal interphalangeal joints of the coolest finger on each hand were recorded, average calculated.	In the iloprost group, mean digital temperature rose from 29.5°C at baseline to 31.6°C at visit 1, then dropped to 28.1°C by visit 4. In the placebo group, it increased from 29.2°C to 30.3°C at visit 1, then fell to 28.8°C by visit 4. ANOVA showed a significant effect of temperature over time but no difference between groups.
		Placebo group	7	59.1	86			
Casas-Barragán et al., 2021^29^	Obs CSS	Patients with FMS	80	30–70	100	FMS	Using a FLIR B335 camera at 20°C and 0.98 emissivity, patients were acclimated for 20 minutes in a 20°C room. The camera, positioned 60 cm from the hypothenar eminence, captured images with hands splayed. Core temperature was measured in the external auditory canal.	Women with fibromyalgia syndrome (FMS) exhibited higher tympanic core and hypothenar eminence temperatures compared to healthy women. These findings showed a correlation between body and hand temperatures and the severity of clinical symptoms in FMS patients.
		Healthy controls	80		100	NA		
Coleiro et al., 2001^73^	RCT	Primary RP patients	26	49.2–56.6	76.92	RP	Using a Starsight Insight Vision Systems camera, baseline thermal images were taken after 15 minutes in a 23°C room. Hands were immersed in 15°C water for 1 minute, with images captured immediately and 10 minutes later. Rewarming was assessed by averaging finger temperatures.	Fluoxetine resulted in significantly greater rewarming post-cold challenge compared to baseline, particularly in females and individuals with primary Raynaud's. Nifedipine showed no significant improvement in rewarming.
		Secondary RP patients	27	50.8–55.6	81.48			
Dziadzio et al., 1999^74^	RCT	Losartan group	26	51	84.62	Patients with primary RP or RP secondary to SSc	Patients acclimated for 15 minutes in a 23°C room. Thermal images of both hands were recorded using a Starsight Thermal Camera before, immediately after, and at 1-minute intervals for 10 minutes post-challenge. Hands were immersed in 15°C water for 1 minute.	Thermography showed no significant recovery 10 minutes after the cold challenge for either treatment group and did not support the clinical improvements reported by patients.
		Nifedipine group	26	51	65.38			
Herrick et al., 2014^44^	RCT	Patients with SSc	12	58	83	SSc	Hand images were captured using an Agema Thermavision 570 camera from 0.5 meters. A baseline image was taken before cooling, followed by a series of four images per minute after cooling.	Subjects received a single oral dose of 30 mg or 100 mg ORM-12741 or placebo at each visit. Temperature recovery after a cold challenge, measured by the AUC of the right index finger, was greater with placebo, with significant differences only for the 30mg ORM-12741 dose.
Martini et al., 2002^66^	RS	Children with JLS	40	5.7	65	SSc	Thermographs were taken with a StarSight pyroelectric infrared imager in a 23°C ± 1°C room after 10–15 minutes of acclimatisation. Lesions were positive if an area was more than 0.5°C warmer than the corresponding opposite limb or body area, or warmer than the surrounding skin if bilateral.	Thermography demonstrated a sensitivity of 92% and a specificity of 68% in detecting disease activity. There was full concordance with clinical reports in 91% of active lesions and 68% of inactive lesions. For lesions examined within two years of onset, thermography's specificity increased to 87.5%, indicating greater reliability in detecting activity in newer lesions.
Martini et al. 2019^45^	CSS	Patients with PRP	14	12.2	71.4	PRP	Thermographic images were captured with a ThermaCAM PM695 after 20 minutes at 23 ± 2°C. Hands, immersed in 15°C water for 1 minute with latex gloves, were imaged pre-test and at 1-minute intervals for 10 minutes during rewarming. Two physicians measured temperatures at the MCP and DIP joints of fingers II–V.	Patients with PRP, SRP, and AC had lower temperatures at DIP and MCP joints compared to controls. During rewarming, these patients showed slower temperature recovery than controls. PRP patients exhibited a faster and greater temperature gain, especially at DIPs, and returned to baseline temperatures by the end of rewarming, unlike SRP and AC patients. The analysis confirmed significant differences in recovery patterns among controls and PRP, SRP, and AC patients
		Patients with SRP	16	11.9	68.8	SRP		
		Patients with AC	14	14.2	50	AC		
		Control group	15	12.4	80	NA		
Miziołek et al., 2021^67^	Obs	Patients with SSc	19	58		SSc	Participants acclimated for 15 minutes at 21°C and 50–60% humidity. Using a FLIR T420 camera, images were taken from 0.6 meters. Temperatures were measured at two ROIs: 10 mm diameter over the nailfold and fingers II–V, and 30 mm diameter at the metacarpus centre.	There was a moderate correlation between thermographic parameters and capillary density in fingers. The early pattern was associated with significantly higher surface temperatures (Tavg) of nailfolds and milder ΔTavg in fingers II-V compared to other capillaroscopic patterns. In fingers, II–V with a higher risk of developing digital ulcers (DU), Tavg was significantly lower and ΔTavg was more pronounced
Miziołek et al., 2023^68^	Obs	lcSSc group	29	55	72.4	SSc	Using a FLIR One Pro camera on an iPhone 11, images were taken at 21°C and 50–60% humidity after 15 minutes of acclimatisation. Images were captured from 0.4 meters with two ROIs: 10 mm over the nailfolds and 30 mm over the dorsal metacarpus. Tavg and TΔ were calculated.	dcSSc patients had cooler fingers and greater temperature gradients than lcSSc patients. Among lcSSc patients, ACA positivity was linked to cooler fingers than anti-Scl70 positivity. While temperature measurements were similar between SSc patients and healthy controls, SSc patients had significantly greater TΔ compared to controls.
		dcSSc group	10	54	60			
		Healthy controls	10	Age-matched	NR	NA		
Schlager et al., 2010^71^	CCS	Patients with RP	25	43.9	NR	RP	Participants acclimated for 20 minutes at 23.3 ± 0.6°C. Using a Thermo Tracer TH1100 IRT device, temperature maps of the left hand's volar surface were recorded before and after 1-minute immersion in 20°C water with gloves. Fingertip temperatures were also measured.	Significant correlation was found between IT and LDPI in both primary RP patients and healthy controls at baseline and after cold challenge. A significant correlation between IT and LDPI changes after cold provocation was found among Raynaud's patients but not in controls
		Healthy controls	22	40.9	NR	NA		
Schuhfried et al., 2000^46^	CSS	Patients with and without definite RP*	86	45.5	84%	Connective tissue diseases**	IRT was conducted in a 24°C room after 20 minutes of acclimatisation. A 1-minute cold-water test at 16°C was performed with latex gloves. Thermograms were captured before, immediately after, and up to 20 minutes post-challenge at points on the fingers and hands.	The LTDpre effectively discriminated between patients with and without Raynaud's phenomenon. Higher likelihood of RP correlated with more negative LTDpre values. LTDpre correctly classified 22 of 23 subjects without clinical RP and 20 of 26 patients with definite RP. However, those with possible or probable RP were often misclassified.
Sempere-Rubio et al., 2021^30^	CSS	Women with FMS	86	54.91	100	FMS	Thermal images were captured with a FLIR E60BX camera (320 × 240, 60Hz) positioned 1 meter away on a tripod, perpendicular to the neck, upper back, lower back, chest, knees, and elbows, with an emissivity of 0.98 Ɛ.	There were no significant skin temperature differences between women with fibromyalgia and healthy controls in the neck, upper back, chest, and elbows. Although significant differences were observed in the lower back and knees, these did not meet the minimum clinically detectable change of 0.5°C.
		Healthy women	92	54.89	100	NA		
Kümpel et al; 2023^31^	CSS	FMS group	80	54.8	100	FMS	The thermography protocol used a T-Series Ultimate camera with high resolution and sensitivity. FLIR T640 cameras captured images in a controlled room at 23°C, 45% humidity, and <0.2 m/s air speed. A trained evaluator took four images using a rainbow scale (23° to 35°C, 0.98 emissivity) following American Academy of Thermology guidelines.	Thermography was not found to be very sensitive or specific for diagnosing fibromyalgia or detecting pain at tender points. The analysis using Receiver Operating Characteristic curves showed that the area under the curve was equal to or lower than 0.75 for all tender points evaluated.
		Control Group	23	51.4		NA		
Sternbersky et al., 2021^47^	RCS	Patients with a history of cold hands	150	41.1	76%	NR	With a Flir B-360 camera, baseline thermograms were taken after 30 minutes in a 22.3°C, 50% humidity room. Hands were immersed in 8.8°C water for 5 minutes, followed by thermograms at 0, 5-, 10-, 20-, and 30-minutes post-immersion.	Out of the 150 patients evaluated with infrared thermography (IRT), 127 were diagnosed with RP, 7 with acrocyanosis, and physiological findings were identified in the remaining 16 patients.
Wilkinson et al., 2018^72^	MCOS	Patients with SSc-related RP	159	63.3	77	SSc-related RP	Patients’ hands were imaged at baseline, then immersed in 15°C water for 1 minute. Rewarming was monitored for 15 minutes with standard thermography capturing 4 frames per minute and mobile thermography recording images at baseline, immediately post-cooling, and 15 minutes post-cooling.	The area under the curve and maximum temperature measures demonstrated strong test-retest reliability. Additionally, there was high convergent validity between thermography and laser speckle contrast imaging.
Zulian et al., 2011^70^	RCT	Patients treated with MTX	46	9	74	JLS	IRT with ThermaCAM PM695 assessed inflammation in skin lesions. Patients were scanned 15 minutes after acclimatisation. Lesions were deemed active if ≥0.5°C warmer than the corresponding site on the opposite side of the body.	ΔTh% decreased significantly more in the methotrexate group compared to placebo (−44.4% vs −12.1%). In the methotrexate group, mean Th% decreased by 49% at 3 months and 44% at 12 months, while in the placebo group, Th% decreased by 50% at 3 months but only 12% at 12 months.
		Placebogroup	24	10.2	67			

* Patients referred from the Division of Rheumatology consecutively for verification or exclusion of secondary Raynaud’s phenomenon,

** 17 with progressive systemic sclerosis, 3 with CREST syndrome (calcinosis, Raynaud’s phenomenon, oesophageal motility disturbances, sclerodactyly, telangiectasia), 7 with SLE, 8 with mcp’s syndrome, 2 with mixed connective tissue disease, 3 with rheumatoid arthritis. Seven patients were suspected of having a connective tissue disease because of a positive test for antinuclear antibodies. Twenty-one patients suffered from polyarthralgia. Eighteen subjects had various diagnoses, for example: inflammatory bowel disease, psoriasis, or carpal tunnel syndrome.

Obs: Observational; RS: Retrospective study; RCS: Retrospective cohort study; MCOS: Multicentre Observational Study; CSS: Cross-sectional study; CCS: Case-control study; SS: Sjögren's syndrome; EDE: Evaporative dry eyes; ADDE: Aqueous deficient dry eyes; JLS: juvenile localised scleroderma; IRT: Infrared thermography; FMS: Fibromyalgia Syndrome; MCP: Metacarpophalangeal; RP: Raynaud's phenomenon; PRP: Primary Raynaud's phenomenon; SRP: Secondary Raynaud's phenomenon; AC: Acrocyanosis; Tavg: average temperature; lcSSc: Limited cutaneous Systemic Sclerosis; SSc: Systemic sclerosis; dcSSc: diffuse cutaneous disease; TΔ: temperature gradients; RCT: Randomised controlled trial; MTX: Methotrexate.

### Diagnostic applications

#### Sjogren’s syndrome

According to Abreau et al., IRT can differentiate dry eye aetiologies and provide insights into the pathophysiological changes causing ocular surface temperature (OST) alterations. The study found that dry eye groups had cooler ocular surface temperatures than normal eyes, with correlations between ocular surface temperature, eyelid temperature, tear film breakup time, and cooling rates, suggesting a link between lid/periorbital temperatures and tear film characteristics in dry eyes. Relative to normal eyes, dry eyes demonstrated significantly lower initial central OSTs and central upper lid temperatures (P<.0001). Initial OST was positively correlated with Tear Film Break-Up Time (TFBUT) (Spearman r = 0.3370, P<.05) and initial time-segmented cooling rate (Spearman r = 0.3992, P=.01).^24^

#### Osteoarthritis

Alfieri et al. found that individuals with osteoarthritis (OA) exhibited higher temperatures in the affected knee, with a statistically significant difference between the affected (29.5 ± 1.4°C) and the unaffected knees (29.2 ± 1.2°C) (P = 0.03). However, there were weak, non-significant correlations between knee temperature and other study variables.^25^ Arfaoui et al. demonstrated that IRT is a reliable diagnostic tool for detecting quantifiable skin temperature patterns in participants with OA. Participants with OA reported an average pain score of 2.5 on a 0–4 scale. The study found that temperature variations correlated with changes in pain intensity for the OA group. Healthy knees averaged 25.83°C, while knees with OA averaged 28.75°C, showing a difference of nearly 2°C.^26^ Bhowmik et al. demonstrated that IRT is effective in detecting inflammation associated with arthritis. The study highlighted IRT’s ability to differentiate between OA and rheumatoid arthritis (RA) based on distinct thermal patterns.^27^ De et al. studied sixty symptomatic knee OA patients, finding that IRT could identify different OA patterns and correlate temperature changes with demographic and clinical characteristics. The mean VAS pain score was 5.6 at the time of imaging. Lower VAS pain scores were associated with greater temperature changes. Higher International Knee Documentation Committee subjective scores correlated with increased temperature variations. Additionally, higher Knee Injury and Osteoarthritis Outcome Score Activities of daily living and Sport/Rec subscale scores were linked to larger temperature changes, while higher PainDETECT scores corresponded with smaller temperature variations.^28^

#### Fibromyalgia

Casas-Barragán et al. highlighted the potential of thermography in assessing clinical symptoms in fibromyalgia patients. The findings demonstrated significant correlations between body core temperature, hand temperature, and the severity of clinical symptoms in women with fibromyalgia. Significant associations were observed between overall impact (β = 0.033; P = 0.030), daytime dysfunction (β = 0.203; P = 0.039), and reduced activity (β = 0.045; P = 0.029) with core body temperature in women with fibromyalgia syndrome. Additionally, pressure pain thresholds at the greater trochanter, both dominant (β = 0.254; P = 0.047) and non-dominant (β = 0.650; P = 0.013), as well as the use of sleeping medication (β = −0.242; P = 0.039), were associated with hypothenar eminence temperature.^29^ In contrast, Sempere-Rubio reported that IRT is not an effective supplementary assessment tool in women with fibromyalgia, as it showed no clinically meaningful reduction or difference in skin temperature at rest when compared with a group of healthy women.^30^ A cross-sectional study by Kümpel et al. evaluated the sensitivity and specificity of computerised IRT as a diagnostic tool for fibromyalgia in 103 participants. The results showed that thermography exhibited low sensitivity and specificity for detecting pain at tender points, with an area under the curve equal to or lower than 0.75, suggesting that thermography is not a reliable diagnostic method for fibromyalgia.^31^

#### Rheumatoid arthritis

Frize et al. reported a significant difference between the temperatures of certain joints in RA patients and the normal (control) group. The best joints to measure were the metacarpals of the hand, specifically the 2nd and 3rd joints.^32^ Gatt et al. (2019) observed significant differences in the mean temperatures between the palm and finger regions of healthy individuals and those with RA. In healthy participants, the mean palm temperature was 29.37°C, and the mean finger temperature was 27.16°C. Conversely, participants with RA showed higher temperatures, with mean palm temperatures of 31.4°C and mean finger temperatures of 30.22°C.^33^ In another study, Gatt et al. (2020) found that RA patients in clinical and radiological remission displayed distinct thermographic patterns in their feet compared to healthy controls, even in unaffected joints. The mean temperatures recorded were as follows: lateral forefoot 27.86°C in RA patients versus 26.79°C in controls, central forefoot 27.87°C in RA patients versus 26.78°C in controls, and medial forefoot 27.91°C in RA patients versus 26.78°C in controls.^34^

Similar findings were reported by Gizińska et al., who demonstrated that thermal imaging, through whole-image observation and temperature distribution analysis, can aid in diagnosing RA and evaluating disease progression. Out of the total joints examined, 183 (22.59%) were found to be inflamed, while 235 joints (29.01%) were reported as painful, and 67 joints (8.27%) exhibited swelling. The RA group consistently showed higher average surface temperatures across all measured areas. The temperature range for the RA group was 30.68°C to 31.71°C, compared to 29.12°C to 30.02°C for the control group.^35^ Snekhalatha et al. showed that both hands of all patients were symmetrically affected by RA, with mean skin temperatures significantly higher in RA patients compared to controls. The Metacarpophalangeal 3, Proximal interphalangeal 3, and distal interphalangeal joint (DIP) 3 joints showed significant temperature increases of 5.3%, 4.9%, and 4.8%, respectively. Additionally, the Heat Distribution Index in RA patients was 51.89% higher than in controls, and the thermographic index was 35.64% greater in RA patients compared to the normal group.^36^

Morales-Ivorra showed that the Thermographic Joint Inflammation Score (ThermoJIS), derived from thermal imaging, effectively detected active synovitis (Area Under the Receiver Operating Characteristic curve: 0.78 (95% CI, 0.71 to 0.86) in patients with RA. ThermoJIS correlated moderately with ultrasound measures of inflammation, including grey-scale synovial hypertrophy (GS, Spearman’s rho = 0.49) and Power Doppler (PD, rho = 0.51). ThermoJIS also showed weaker correlations with clinical and laboratory markers such as swollen joint count (SJC28, rho = 0.38), tender joint count (TJC28, rho = 0.33), C-reactive protein (CRP, rho = 0.20), and erythrocyte sedimentation rate (ESR, rho = 0.28). In the validation set, 41.1% of patients were in remission based on Disease Activity Score (DAS)28-CRP criteria (<2.6), 19.9% based on CDAI (≤2.8), 20.5% based on SDAI (≤3.3), and 16.4% based on the ACR/EULAR Boolean definition of remission ^37^. Umapathy et al. found thermography to be an effective tool for early detection of RA in the knee, with a computer-aided model serving as a potential pre-screening method. The average temperature difference between RA patients with a mean DAS of 4.9 and healthy individuals was 5.94%. RA patients had an average skin temperature of 33.99 ± 0.9°C, compared to 31.97 ± 0.7°C for normal subjects. Correlations with skin temperature included Health Assessment Questionnaire score ((r = 0.75), DAS score (r = 0.70), ESR (r = 0.75), and CRP (r = 0.64).^38^

Vasdev et al. identified significant differences in knee temperature and the knee-thigh temperature differential in RA patients (76% of patients had moderate disease activity, 22% had low activity, and 2% had high activity) with inflamed knee joints. The mean knee temperatures were lower in the control group (31.490°C) compared to RA patients (32.732°C). The temperature differential between knees and thighs was also significantly smaller in controls (−0.5°C) than in RA patients (+1.08°C).^39^

#### Juvenile idiopathic arthritis

Lerkvaleekul et al. highlighted the efficacy of IRT in assessing wrist arthritis in patients with juvenile idiopathic arthritis (JIA). The study found that mean temperatures (T_mean) for healthy controls, inactive arthritis, mild arthritis, and moderate to severe arthritis groups were 30.27°C, 30.17°C, 31.37°C, 31.02°C, and 32.54°C, respectively. Maximum temperatures (T_max) were similar, with values of 30.98°C, 31.07°C, 32.25°C, 32.25°C, 31.84°C, and 33.59°C for the same groups. Significant temperature differences were noted between arthritis groups and healthy controls. IRT also showed strong diagnostic performance for moderate to severe arthritis: T_mean ≥ 31.0°C yielded 85.7% sensitivity and 80.0% specificity (AUC 0.93), while T_max ≥ 32.3°C provided 71.4% sensitivity and 93.3% specificity (AUC 0.91).^40^ Nwaizu et al. found that patients in 20 JIA with active knee inflammation showed elevated temperatures compared to those without. Eight participants had one knee affected, eight had both knees involved, and four had no active arthritis. Knees with active inflammation had a median temperature 3.198% higher than inactive knees.^41^

Lasanen et al. demonstrated thermal imaging may have potential for detecting joint inflammation in ankle joints of children with JIA or autoimmune diseases with arthritis. The surface temperatures of inflamed ankle joints were significantly higher than those of non-inflamed joints. Specifically, the mean temperature difference was 0.503°C greater in inflamed ankles. Additionally, the maximum temperature difference was 0.319°C higher in inflamed ankles.^42^

#### Scleroderma

Agazzi et al. reported that IRT is a valuable tool for detecting disease activity and reliably monitoring lesion progression over time in patients with juvenile localised scleroderma (JLS).^43^ IRT may also help objectively assess peripheral microvascular function in patients with systemic sclerosis (SSc). Herrick et al. reported IRT was successfully used as one of three methods to objectively measure peripheral blood flow changes during cold challenge testing (2014).^44^

#### Raynaud’s phenomenon

Martini et al. found IRT to be a reliable and reproducible method (Intraclass correlation coefficient > 0.93) for assessing peripheral microvascular disturbances in children. IRT effectively distinguished primary Raynaud’s phenomenon (PRP), secondary Raynaud’s phenomenon (SRP), and acrocyanosis through differences in baseline temperatures and rewarming patterns. PRP patients showed higher DIP temperatures (29.96°C in PRP vs. 29.31°C in SRP and 25.66°C in acrocyanosis) and smaller distal-dorsal differences (0.56°C in PRP vs. 1.99°C in SRP). After a cold challenge, PRP patients demonstrated faster and more complete temperature recovery.^45^ Schuhfried et al. showed that IRT can assist in diagnosing secondary RP by effectively discriminating between patients with and without definite RP based on the longitudinal temperature difference before cold challenge test (LTDpre). In patients without Raynaud’s phenomenon, the thermographic method employing the LTDpre demonstrated a sensitivity of 96% and a specificity of 62%. For those with definite Raynaud’s phenomenon, sensitivity was 77% and specificity was 73%. In patients with unlikely or probable Raynaud’s phenomenon, sensitivity dropped to nearly zero (5%), while specificity remained high at 100% and 95%.^46^

Sternbersky et al. illustrated that IRT serves as a diagnostic tool that can differentiate between healthy patients and those with RP.^47^ Lindberg et al. found that at a 0.05 cut-off level, the thermographic algorithm achieved a sensitivity of 65%, specificity of 58%, and accuracy of 66%, demonstrating non-inferiority compared to the finger systolic pressure (FSP) test (sensitivity of 77%, specificity of 37%, and accuracy of 59%). Their findings underscored the utility of thermography in detecting RP and suggested its potential as a replacement for the FSP test in diagnostic settings.^48^

### Monitoring, evaluation, and therapeutic applications

#### Osteoarthritis

Denoble et al. found a significant correlation (R = 0.594) between the cutaneous temperature of the patella and the severity of knee OA. Their study suggests that skin temperature in the patellar region is linked to X-ray severity of knee OA, indicating that infrared knee imaging can serve as a reliable, objective measure of inflammation and its relationship to structural damage in knee OA.^49^ Varjú et al. suggested that thermography could be a non-radioactive, non-invasive method for assessing and monitoring nodal OA. Their study found that mean joint temperature decreased with increasing severity of radiographic OA (rOA) as defined by the Kellgren–Lawrence (KL) scale. A strong correlation was noted between rOA severity and joint surface temperature (P < 0.001). KL1 showed an increase of +0.168°C (P = 0.01), while KL3 exhibited a decrease of −0.202°C (P = 0.04), with no significant changes in KL2 and KL4. The technique demonstrated no significant temperature differences related to Nonsteroidal anti-inflammatory drugs use.^50^

Zhang et al. found that patients with moderate to severe knee osteoarthritis (KOA) had significantly higher knee temperatures than those with mild KOA (p<0.05). A positive correlation was observed between knee temperature and cartilage wear grade from ultrasound (r=0.426). Additionally, knee temperature correlated positively with VAS pain scores (r=0.403) and the Western Ontario and McMaster Universities Osteoarthritis Index (WOMAC) osteoarthritis index (r=0.382).^51^ De et al. observed that patients with poorer clinical status showed a lower temperature response to exercise, and women had a greater temperature decrease than men. According to the study, knee temperature decreased significantly after exercise, from 32.13 ± 1.07°C at baseline (T0) to 31.86 ± 1.12 °C (T1) and 31.94 ± 1.10 °C (T2) (P = 0.002). Women experienced a greater temperature reduction than men at T1 (ΔT0-T1: −0.47 ± 0.64 vs. −0.16 ± 0.49, P = 0.021) and T2 (ΔT0-T2: −0.46 ± 0.49 vs. −0.05 ± 0.59, P = 0.009). Similar trends were observed in the medial and suprapatellar regions.^28^

Brito et al. noted that older adults with OA exhibited distinct skin temperature patterns compared to controls during exercise. In men with OA, thigh temperatures were markedly elevated (anterior: +1.2°C to +1.3°C; posterior: +1.7°C to +1.9°C higher than controls), while knee temperatures were lower (−0.5°C to −0.7°C compared to controls). Women displayed similar but more subtle patterns, with thigh temperatures showing slight elevations (+0.1°C to +0.4°C) and knee temperatures marginally lower (−0.2°C) compared to controls.^52^ De Marziani et al., in a study of 40 patients with symptomatic knee OA, highlighted the clinical value of IRT. They found that higher knee temperatures correlated with younger age, male sex, higher body mass index, and worse knee scores, while lower temperatures were linked to neuropathic pain.^53^ Hegedus et al. (2009) found that IRT appears to be a useful objective tool for assessing microcirculatory changes and inflammatory responses during low-level laser therapy treatment in knee OA patients.^54^

#### Rheumatoid arthritis

Pauk et al. found that IRT with cold provocation testing offers a non-invasive, quantitative method for detecting inflammation in RA patients with moderate to high disease activity. The technique showed promise for assessing the thumb, index, middle and ring fingers, with the index finger demonstrating the highest diagnostic accuracy (ROC AUC = 0.68).^55^ Another study by Pauk et al. demonstrated that in moderate disease activity (n = 16), temperature change during rewarming showed a strong negative correlation with the DAS28 (R = −0.97, P <0.05), number of tender joints (R = −0.96, P <0.05), and mean post-rewarming temperature (R = −0.92, P <0.05). In high disease activity (n = 50), post-cooling temperatures correlated with rheumatoid factor (RF) (R = 0.72, P <0.05) and anti-CCP antibodies (R = 0.75, P<0.05). Temperature change during rewarming (ΔTR) was 1.8–2.6°C for high activity, 3.4–3.9°C for moderate activity, and higher in healthy controls. The area under the heating curve (SR) ranged from 316.7–422.3°C for high activity, 511.6–580.9°C for moderate activity, and 688.1–731.9°C for healthy controls. ^56^

Tan et al. demonstrated a significant correlation between thermographic parameters of IRT and ultrasound-detected inflammation, highlighting its utility in detecting and monitoring synovitis in RA. In a study of 37 RA patients (DAS28 score: 4.43), PD-positive joints showed higher thermographic temperatures compared to PD-negative joints, with a maximum temperature difference of 1.37°C (32.5°C vs. 31.1°C), a minimum difference of 0.91°C (30.2°C vs. 29.3°C), and an average difference of 1.16°C (31.3°C vs. 30.1°C).^57^ The combination of thermography and ultrasound imaging was found to be more effective than using either technique alone, based on its correlation with the DAS-28 in RA patients (DAS28 score: 4.43). Correlation coefficients for MAX PD and AVG PD were r = 0.393 (P = 0.016) and r = 0.376 (P = 0.022), respectively (Tan et al., 2021).^58^ Another study by Tan et al. (2024) on RA patients (DAS28 score: 3.83) found significant correlations between thermographic temperatures and ultrasound greyscale scores at the elbow, with coefficients of 0.39 (MIN), 0.40 (MAX), and 0.42 (AVG), reinforcing thermography’s role in inflammation assessment.^59^

Devereaux et al. found significant correlations between thermography and clinical parameters such as ESR, Ritchie articular index, grip strength Mallya score, morning stiffness, and pain score (r= 0.280, 0.685, −0.462, 0.547, 0.394, and 0.255), validating the use of the summated Heat Distribution Index for assessing therapy response in RA patients.^60^ However, Jones et al. indicated that joint thermography does not significantly correlate with other clinical markers of disease and appears to be ineffective for assessing small joints of the hand in inflammatory arthritis.^61^ Vinson et al. found that while thermal imaging was not effective for assessing RA activity in small joints, it showed promising temperature variations in inflamed joints in patients with moderate disease activity. Temperature differences between joints and the forearm significantly correlated with tender joints (P < 0.001) and US-detected synovitis in B-mode (P = 0.021).^62^ Goldie et al. reported that thermography shows promise in effectively evaluating the efficacy of anti-inflammatory drugs like naproxen, by detecting rapid vascular changes associated with inflammation in RA patients.^63^

#### Juvenile idiopathic arthritis

Zhao et al. using a novel infrared thermal imaging algorithm showed higher temperature after within-limb calibration (TAWiC) in children with active arthritis or tenosynovitis compared to healthy joints, demonstrating its clinical utility for arthritis detection. Inflamed joints showed higher TAWiC values than non-inflamed ones, with mean TAWiC for knees at 0.67°C (anterior), 0.89°C (medial), and 0.72°C (lateral) compared to −0.39°C, 0.02°C, and −0.13°C in non-inflamed joints, respectively. For ankles (anterior view), inflamed joints had a mean TAWiC of −0.42°C versus −0.84°C in non-inflamed joints.^64^ Balay-Dustrude et al. investigated IRT to detect joint inflammation in children with JIA. The researchers utilised the TAWiC algorithm and reported 80% sensitivity and 84.2% specificity in identifying active joint inflammation from anterior knee views.^65^

#### Scleroderma

Martini et al. showcased that thermography, when combined with clinical examination, serves as a promising tool for assessing disease activity. This approach is particularly effective as long as the lesions do not exhibit severe skin and subcutaneous fat atrophy. The study revealed that thermography achieved a sensitivity of 92% and a specificity of 68%.^66^ Miziołek et al. highlighted IRT as a promising non-invasive tool for assessing microvascular abnormalities, disease progression, and predicting digital ulcer risk in SSc patients, complementing nailfold videocapillaroscopy. A moderate positive correlation (r = 0.5, P < 0.001) was found between nailfold temperature and capillary density in fingers II–V, with the strongest correlations observed in the fourth (r = 0.61) and fifth (r = 0.55) fingers. Notably, fingers with high risk of digital ulcers (capillaroscopic skin ulcers risk index ≥ 2.96) exhibited significantly lower nailfold temperatures (26.7 ±2.9°C) compared to low-risk fingers (29.0 ±2.7°C, P < 0.001).^67^

Thermographic analysis revealed distinct thermal patterns across SSc subtypes, with diffuse cutaneous systemic sclerosis (dcSSc) patients showing significantly impaired thermal control compared to limited cutaneous systemic sclerosis (lcSSc). This was reflected in lower average temperatures in thumbs (26.7°C vs. 30.5°C, P <0.001) and fingers II–V (24.0°C vs. 28.6°C, P <0.001), alongside more pronounced temperature gradients in thumbs (2.5°C vs. 1.1°C, P = 0.03) and fingers II–V (6.3°C vs. 3.0°C, P <0.001). Among lcSSc patients, ACA positivity was associated with lower finger temperatures (28.2°C vs. 29.3°C, P = 0.033) and greater temperature gradients (3.6°C vs. 2.2°C, P = 0.025) compared to anti-Scl70 positivity, though this difference was significant only for fingers II–V. (Miziołek et al., 2023). ^68^ Bali et al. utilised thermography to quantify changes in digital temperature before and after iloprost or placebo infusions over 4 months in patients with RP and SSc.^69^ Zulian et al. demonstrated the effectiveness of IRT in correlating with disease activity in JLS, identifying active lesions by elevated temperatures, with MTX-treated patients showing a 44.4% mean temperature reduction versus 12.1% in the placebo group (P = 0.024).^70^

#### Raynaud’s phenomenon

Schlager et al. reported IRT as a non-invasive technique to assess skin perfusion and vasoreactivity in RP. They found a strong correlation between IRT and laser Doppler perfusion imaging (LDPI) measurements in both Raynaud’s patients and healthy controls. At baseline (room temperature), Raynaud’s patients showed mean fingertip temperatures of 31.2 ± 3.7°C compared to 35.42 ± 3.1°C in healthy controls. The correlation was particularly strong in Raynaud’s patients (r= 0.868) compared to healthy controls (r= 0.790).^71^ Wilkinson et al. recommended using thermography as a secondary outcome measure in clinical trials, focusing on the area under the curve (AUC) and maximum temperature (MAX) as key parameters for evaluating treatment efficacy. This recommendation was supported by their findings showing substantial test-retest reliability for both thermography measurements [AUC intraclass correlation coefficients (ICCs) = 0.68; MAX ICC = 0.72] and strong convergent validity with laser speckle contrast imaging (latent correlations: AUC ρ = 0.94; MAX ρ = 0.87).^72^

Coleiro et al. showed that IRT objectively assessed treatment responses in RP, revealing significant improvements in digital rewarming with fluoxetine treatment, especially in females and PRP patients. After fluoxetine treatment, female patients showed significant improvement in rewarming (from 29.0% to 44.6%). Patients with PRP demonstrated the most dramatic improvement with fluoxetine, with rewarming increasing from 33.4% to 58.8% (P = 0.03). In contrast, those with SRP showed minimal change (31.6% to 31.2%).^73^ However, Dziadzio et al. reported that thermography did not demonstrate any significant improvement in vascular response or hand temperature recovery after cold challenge in patients treated with losartan or nifedipine in patients with PRP or RP secondary to SSc.^74^

### Detection of subclinical disease

Morales-Ivorra et al. demonstrated that ThermoJIS could identify patients with persistent subclinical joint inflammation, indicating an elevated risk of flares and structural damage progression.^37^ Similarly, Gatt et al. found that thermal patterns distinguished RA patients from controls, even in remission, suggesting that IRT could detect subclinical disease.^34^ Tan et al. (2021) reported that combining maximum and average temperature parameters with ultrasound strongly correlated with DAS.^58^

Varjü et al. found that higher joint surface temperatures in early radiographic OA stages indicated the potential of IRT for early detection.^50^ Zhang et al. showed that knee temperature correlated with cartilage wear and pain scores, suggesting its potential to detect physiological changes before structural damage.^51^ Alfieri et al. noted significant temperature differences, with affected knees showing 0.3°C higher temperatures than unaffected ones,^25^ while Arfaoui et al. reported that IRT revealed asymmetrical thermal patterns and correlations with pain intensity, enabling detection before radiographic changes appeared.^26^

Vasdev et al. highlighted the high sensitivity and specificity of thermal imaging in detecting joint inflammation.^39^ Miziołek et al. utilised IRT to evaluate micro-vasculopathy progression in SSc, correlating changes with nailfold capillaroscopy patterns.^67^ Additionally, Lasanen et al. found that IRT showed elevated temperatures in inflamed ankle joints compared to non-inflamed ones, supporting its utility in paediatric cases.^42^ Nwaizu et al. demonstrated that IRT accurately identified temperature differences in inflamed knees, showcasing its reliability in detecting JIA.^41^

### Clinical value of IRT in synovitis assessment

IRT offers several practical advantages compared to gold-standard imaging methods such as ultrasound and Magnetic Resonance Imaging (MRI). It is affordable, easy to operate, and excels in rapid image capturing, making it particularly valuable in clinical settings. Medical professionals can implement IRT with limited training, in contrast to ultrasound, which requires extensive expertise and considerable time investment for joint examinations. Unlike MRI, which poses challenges due to pacemaker restrictions and high costs, IRT is an accessible and mobile alternative suitable for routine monitoring.^37,58^ In the context of RA, both thermography and ultrasound contribute uniquely to understanding synovitis. Thermography provides precise temperature measurements across joint surfaces, allowing for the detection of inflammation patterns, while ultrasound offers detailed information about synovial inflammation and blood flow. The integration of both imaging approaches offers a richer understanding of synovitis and disease progression than either method could achieve independently.^57^

The detailed comparison of IRT, ultrasound, and MRI for synovitis assessment is presented in **Table 3**.^37,57,58,75–78^

**Table 3.** Comparison of IRT, ultrasound, and MRI for synovitis assessment.

**Characteristics**	**IRT**	**Ultrasound**	**MRI**
**Imaging principle**	Assesses skin surface temperature changes	Displays the synovial structures and the flow of blood	Imaging of soft tissues and inflammation at a high resolution
**Current role**	Emerging complementary tool for routine practice	Established standard practice tool	Gold standard for comprehensive assessment
**Clinical capabilities**	Detection of warmth as sign of inflammation	Visualising synovitis, tenosynovitis, and enthesitis, detecting bone erosions and crystal deposits, and assessing soft tissue and vascularity	Enhanced imaging delivers superior soft tissue contrast and detailed 3D visualisation, effectively detecting bone marrow oedema and quantifying synovial processes
**Operator dependence**	Minimal (minimal training required)	High (involves expertise for interpretation)	Low (automated imaging, although interpretation is necessary)
**Portability**	High (compact, easy to use)	Moderate (requires specialised equipment)	Low (requires large, immobile equipment)
**Cost**	Low	Moderate (Relatively lower cost than MRI)	High
**Time proficiency**	Fast (Quick image acquisition)	Moderate	Slow (lengthy acquisition and analysis time)
**Limitations**	Only surface temperature assessment, cannot visualise underlying structures, and limited validation compared to other methods	Operator dependent, requires substantial training, time-consuming for multiple joints, and cannot assess beyond bony cortex	High cost, limited availability, contraindications (e.g., pacemakers), and less feasibility for routine monitoring

## DISCUSSION

Regarding the diagnostic application, the systematic review yielded one article for Sjogren’s syndrome, four for OA, and three for fibromyalgia.^24–31^ However, there are limited studies on its clinical use for diagnosing dry eye conditions in Sjogren’s. A case study by Luong et al. showed that using IRT at different ambient temperatures improved the definition of thermoregulatory sweating abnormalities in a patient with Ross syndrome associated with Sjogren’s.^79^

With regard to OA, IRT can differentiate between unilateral inflammation seen in OA and bilateral inflammation in RA. The non-intrusive nature of IRT allows for early detection of OA and continuous monitoring of its progression. A study by Calin et al. indicated that thermal infrared imaging could serve as a valuable complementary method alongside conventional imaging techniques such as X-rays, ultrasound, and MRI for assessing degenerative and inflammatory knee pathologies. It was found to enhance diagnostic accuracy and improve clinical and functional follow-up of patients, with broad application potential in rheumatology, orthopaedics, and rehabilitation clinics.^80^ The present systematic review has yielded mixed results with regard to the use of IRT for the diagnosis of fibromyalgia.^29–31^

For the diagnostic application with reference to RA, JIA, scleroderma, and RP, the number of articles considered by the present review was seven, three, two, and four respectively. In RA, IRT helps in identifying temperature differences in joints, aiding early detection and management of RA, even in remission. It is also effective in diagnosing and monitoring disease progression through methods like whole-image observation and ThermoJIS.^32–35^ A study by Kow et al. reported that thermal imaging was a relatively low-cost, non-invasive technique with high feasibility due to its short image acquisition time, portability, and ease of setup. It provided an objective assessment of joint surface temperature, serving as an adjunctive tool in evaluating joint inflammation in patients with RA.^81^

The current review showed similarly promising results for JIA. IRT effectively differentiates active from inactive arthritis in JIA, offering a non-invasive and child-friendly monitoring method.^40,41^ The present review also highlighted the clinical utility of IRT in detecting disease activity and assessing peripheral microvascular function in patients with scleroderma.^43,44^ For RP, studies also emphasised the ability of IRT to identify significant differences in extremity temperatures and rewarming patterns, enhancing diagnostic accuracy and potentially replacing more invasive tests.^45–48^

With reference to monitoring and evaluation, the current review considered six studies each for OA and RA. IRT in OA correlates with inflammation, structural damage, and pain severity, providing a non-invasive tool for early assessment and treatment monitoring. Studies highlight demographic and clinical influences on skin temperature patterns, with variations observed pre- and post-exercise, particularly by gender and age.^28,49–53^ A systematic review by Schiavon et al. evaluated 32 articles and found a significant association between thermal findings and disease status, as well as the clinical assessment of disease activity and treatment response in inflammatory and degenerative joint diseases. The study has concluded IRT is a simple, accurate, non-invasive, and radiation-free method that can complement existing tools for screening, diagnosis, monitoring disease progression, and evaluating treatment response.^11^

With reference to RA, the findings from various studies highlight the potential of IRT as a valuable tool for assessing inflammation. IRT, when combined with temperature profiling and cold provocation testing, is effective in detecting inflammation and distinguishing RA patients from healthy individuals. Furthermore, IRT shows significant correlation with ultrasound-detected inflammation and is particularly useful for monitoring synovitis in RA. The combination of IRT and ultrasound is more effective than either method alone for assessing disease activity.^55–62^ A mini-review by Fokam and Lehmann noted that several studies reported a significant correlation between IRT-based skin temperature measurement and arthritis knee pain in both OA and RA, concluding it is an effective tool for clinical assessment.^82^ However, there are limitations to the clinical utility of IRT for small joint assessments, and further validation in clinical practice is needed.

For monitoring and assessment, the number of articles considered for JIA, scleroderma, and RP were two, three, and one respectively. Studies have demonstrated the effectiveness of the TAWiC algorithm in detecting inflammation in children, underscoring its utility for early diagnosis and monitoring of active arthritis.^64,65^ In scleroderma, thermography is effective in assessing disease activity, evaluating microvascular abnormalities, predicting digital ulcer risk, and distinguishing thermal patterns in SSc subtypes, suggesting that IRT can complement traditional assessment method.^66–68^ Additionally, IRT is effective in evaluating skin perfusion and vasoreactivity in RP, indicating its non-invasive nature as a promising tool for clinical assessments.^71^ Marek Chojnowski has highlighted the clinical use of IRT in everyday practice with the advent of low-cost infrared cameras. The study underscores its potential in confirming the diagnosis of RP and monitoring disease activity and treatment response in SSc.^83^

Regarding therapeutic applications, the current review considered one study each for OA and RA, and two and three studies for SSc and RP, respectively. IRT holds significant potential as an objective tool for assessing inflammation, microcirculatory changes, and treatment responses across various rheumatic conditions. It has proven effective in evaluating the efficacy of low-level laser therapy in knee OA, anti-inflammatory drug treatments in RA, and digital temperature changes in RP and SSc patients following specific treatments. It is also useful in objectively quantifying lesion activity in JLS and assessing treatment responses in RP.^54,63,69,70,72–74^ Pauling et al. reported that IRT as a microvascular imaging tool holds the potential to circumvent the limitations of self-report assessment of RP.^84^

With recent advancements in affordable infrared cameras, IRT holds promise as an adjunct tool in routine rheumatology practice. It serves as a safe, cost-effective, non-invasive, and radiation-free imaging modality for the early detection of disease, monitoring disease progression and treatment responses, and evaluating microvascular changes, skin perfusion, and rewarming patterns in vascular-related rheumatic conditions. Additionally, it provides an objective method to assess the efficacy of therapies, including anti-inflammatory drugs, low-level laser therapy, and vasoactive agents.^11,56,85^ The adoption of IRT is also revolutionising other medical fields, including oncology, cardiology, dermatology, gynaecology, and fever screening. It aids in diagnosing breast cancer, diabetic neuropathy, peripheral vascular disorders, and other conditions by detecting abnormal heat patterns. IRT is particularly valuable in oncology for early tumour detection, monitoring treatment responses, and assessing post-surgical complications. In cardiology, it assists in diagnosing peripheral artery disease, monitoring vascular inflammation, and evaluating tissue perfusion.^86,87^

However, several limitations impair the wider clinical adoption of IRT. Its increased sensitivity to environmental factors like ambient temperature, humidity, and lighting, can affect accuracy.1 The lack of standardised protocols for image acquisition and interpretation further reduces reliability, while artifacts from patient movement or external heat sources can compromise results. Additionally, the high initial costs of advanced infrared cameras and ongoing maintenance pose significant challenges, especially in resource-limited settings. Specialised training and expertise are required for optimal use, and variability in image interpretation affects diagnostic consistency.^88^ Addressing these challenges through improved technology, cost reduction, standardisation, and targeted training is essential to maximise the clinical utility of IRT.

The current review findings highlight the lack of clear consensus and standard protocol for the clinical use of IRT. Despite its potential in diagnosing and monitoring conditions such as OA, RA, SSc, and RP, standardised guidelines and protocols are necessary to ensure consistent and reliable application in clinical practice. The systematic review exhibits several strengths and limitations. The adoption of methodology adhered to PRISMA guidelines provides a systematic and transparent approach. It employed inclusive criteria without a time limit, maximising the number of relevant publications on the clinical application of IRT in rheumatic diseases. A thorough search strategy was implemented across multiple databases and supplementary sources, enhancing the comprehensiveness of the review. Additionally, a rigorous quality assessment was conducted using standardised tools, ensuring the reliability and validity of the included studies. However, the review also has notable limitations. It only considered articles written in English, potentially excluding relevant studies in other languages. Furthermore, variability in the methodological quality of the included studies was observed, particularly in cross-sectional studies. The lack of a meta-analysis due to data inconsistencies also restricts the ability to quantitatively synthesise findings. While the systematic review provides valuable insights into the clinical applications of IRT in rheumatic diseases, its findings should be interpreted with consideration of these limitations.

IRT effectively differentiates between conditions such as Sjögren’s syndrome, OA, fibromyalgia, RA, JIA, scleroderma, and RP, offering insights into inflammation and disease activity. It serves as a reliable non-invasive method for assessing disease activity and response to treatment, correlating well with clinical parameters, particularly in OA and RA. Additionally, IRT shows promise in evaluating treatment efficacy, especially in RA and scleroderma, by quantifying changes in temperature related to therapeutic interventions. Overall, the integration of IRT into clinical practice for rheumatic diseases could facilitate early diagnosis, improve disease monitoring, and optimise treatment strategies, though further research is needed to standardise protocols and enhance its applicability in diverse clinical settings.

## CONFLICT OF INTEREST

The authors declare no conflict of interest.

## CORRESPONDING AUTHOR’S DECLARATION

I hereby declare that the manuscript is original, unpublished, and not under consideration elsewhere. All authors have contributed substantially, and necessary permissions and acknowledgments are included.

## Figures and Tables

**Supplementary Table 1.** MeSH search terms and corresponding number of articles on infrared thermography in rheumatic diseases.

**MeSH search terms**	**No of articles**
Infrared thermography AND Rheumatic diseases	154
Infrared Camera or Thermal Camera AND Rheumatic diseases	73
Thermography AND Rheumatic diseases	472
Infrared thermography AND Rheumatoid arthritis	206
Infrared thermography AND Osteoarthritis	124
Infrared thermography AND Lupus	47
Infrared thermography AND Psoriatic Arthritis	25
Infrared thermography AND Ankylosing Spondylitis	24
Infrared thermography AND Sjögren’s syndrome	15
Infrared thermography AND Gout	16
Infrared thermography AND Scleroderma	45
Infrared thermography AND Infectious Arthritis	23
Infrared thermography AND Juvenile Idiopathic Arthritis	10
Infrared thermography AND Systemic sclerosis	86
Infrared thermography AND Polymyalgia rheumatica	3
Infrared thermography AND Fibromyalgia	11
Total number of articles	1,334

**Supplementary Table 2.** Quality assessment of cohort studies.

**Study**	**Selection**				**Comparability**		**Outcomes**			**Total quality score**
	**Representativeness of the exposed cohort**	**Selection of the non-exposed cohort**	**Ascertainment of exposure**	**Demonstration that outcome of interest was not present at the start of the study**	**Important factors**	**Other controlled factors**	**Assessment of outcome**	**Follow-up long enough for outcomes to occur**	**Adequacy of follow-up of cohorts**	
Lindberg et al., 2021^48^	*	*	*	**-**	*	**-**	*	**-**	**-**	**5**
Miziołek et al., 2021^67^	*	*	*	*	*	*	*	*	*	**9**
Pauk J et al., 2019^56^	*	*	*	*	*	*	*	*	**-**	**8**
Devereaux et al., 1985^60^	*	**-**	*	*	*	*	*	*	*	**8**
Sternbersky et al., 2021^47^	*	**-**	*	*	*	**-**	*	*	*	**7**
Wilkinson et al., 2018^72^	*	**-**	*	*	*	*	*	*	*	**8**
Zhang et al., 2023^51^	*	*	*	**-**	*	**-**	*	**-**	*	**6**
Balay-Dustrude et al., 2023^65^	*	*	*	*	*	*	*	**-**	**-**	**7**
Brito et al., 2020^52^	*	*	*	NA	*	*	*	*	*	**8**

**Supplementary Table 3.** Quality assessment of case-control studies.

**Study**	**Selection**				**Comparability**		**Outcome**			**Total quality score**
	**Adequacy of the case definition**	**Representativeness of the cases**	**Selection of Controls**	**Definition of Controls**	**Important factors**	**Other controlled factors**	**Ascertainment of exposure**	**Same method of ascertainment for cases and controls**	**Non-Response rate**	
Schlager et al., 2010^71^	*	*	**-**	*	*	*	*	*	**-**	**7**
Sempere-Rubio et al., 2021^30^	*	*	*	*	*	*	*	*	**-**	**8**
Vasdev et al., 2023^39^	*	*	*	*	*	*	*	*	**-**	**8**
Miziołek et al., 2023^68^	*	*	*	*	*	*	*	*	**-**	**8**
Arfaoui et al., 2012^26^	*	**-**	*	*	*	**-**	*	*	**-**	**6**
Gatt et al, 2019^33^	*	*	*	*	*	**-**	*	*	**-**	**7**
Casas-Barragán et al., 2021^29^	*	*	*	*	*	*	*	*	*	**9**
Umapathy 2020^38^	*	*	*	*	*	-	*	*	*	**8**

**Supplementary Table 4.** Quality assessment of cross-sectional studies.

**Study**	**Selection**				**Comparability**	**Outcome**		**Total quality score**
	**Representativeness of the sample**	**Sample size**	**Non-respondents**	**Ascertainment of exposure**	**Based on design and analysis**	**Assessment of the outcome**	**Statistical test**	
Schuhfried et al., 2000^46^	*	*	**-**	**NA**	**	*	*	6
Snekhalatha et al., 2015^36^	*	*	*	**	**	**	*	10
Tan et al., 2020^57^	**-**	**-**	**-**	**	*	**	*	6
Tan et al. 2021^58^	*	**-**	**-**	**	*	**	*	7
Tan et al., 2024^59^	**-**	*	**-**	**	*	*	*	6
Varjú et al., 2004^50^	*	*	**-**	**	**	**	*	9
Vinson et al., 2020^62^	*			**	*	**	*	7
Bhowmik et al.,2016^27^	*	**-**	**-**	**	*	**	*	7
De et al.; 2023^28^	*	*	**-**	**	**	**	*	9
Denoble et al., 2010^49^	**-**	**-**	**-**	**	*	**	*	6
Frize et al., 2011^32^	*		**-**	*	**	*	*	5
Gatt et al., 2020^34^	*	**-**	**-**	**	*	**	**	7
Gizińska et al., 2021^35^	*	*	**-**	**	*	**	*	8
Jones et al., 2018^61^	*	*	**-**	**	*	**	*	8
Lasanen et al., 2015^42^	*	*	**-**	**	*	**	*	8
Lerkvaleekul et al., 2017^40^	*	*	**-**	**	*	**	*	8
Morales-Ivorra et al., 2022^37^	*	*	*	**	**	**	*	10
De Marziani et al; 2023^53^	*	**-**	**-**	*	**	**	*	7
Agazzi et al., 2018^43^	*	*	*	**	**	**	*	10
Abreau et al; 2016^24^	*	*	*	**	*	**	*	9
Zhao et al., 2022^64^	*	*	*	**	**	**	*	10

NA: Not applicable for the study.

**Supplementary Table 5.** QUADAS-2 assessment for risk of bias in diagnostic studies.

**Study**	**Risk of bias**			**Applicability concerns**							
	**Patient selection**	**Index test**	**Reference standard**	**Flow and timing**	**Patient selection**	**Index test**	**Reference standard**
Kümpel et al., 2023^31^	**1**	**3**	**3**	**1**	**1**	**1**	**1**
Martini et al., 2002^66^	**3**	**1**	**1**	**3**	**3**	**1**	**1**
Martini et al., 2019^45^	**3**	**1**	**1**	**3**	**3**	**1**	**1**
Nwaizu et al., 2020^41^	**1**	**3**	**1**	**1**	**1**	**1**	**1**
Pauk et al., 2019^55^	**1**	**1**	**3**	**3**	**1**	**1**	**3**

1= Low risk; 2= High risk; 3= Unclear.

**Supplementary Table 6.** Quality assessment of randomised studies by Jadad score.

**Study**	**Randomised control study**	**Sequence of randomisation described and appropriate**	**Double-blind study**	**Method of double blinding described and appropriate**	**Withdrawals and dropouts**	**Sequence of randomisation described and inappropriate**	**Double-blind method described and inappropriate**	**Jadad scores**
Bali et al., 2011^69^	1	1	1	1	0	0	0	4
Coleiro et al., 2006^73^	1	0	0	0	1	NA	NA	2
Dziadzio et al., 1999^74^	1	0	0	0	1	0	0	2
Goldie et al., 1974^63^	1	0	1	1	1	0	0	4
Hegedus et al., 2009 [54]	1	1	1	1	1	0	0	5
Herrick et al., 2014^44^	1	1	1	1	1	0	0	5
Zulian et al., 2011^70^	1	1	1	1	1	0	0	5

**Supplementary Table 7.** JBI critical appraisal checklist for case series.

**Study/Author**	**Criteria**	**Addressed**
Alfieri et al., 2020^25^	1. Were there clear criteria for inclusion in the case series?	Yes
	2. Was the condition measured in a standard, reliable way for all participants included in the case series?	Yes
	3. Were valid methods used for identification of the condition for all participants included in the case series?	Unclear
	4. Did the case series have consecutive inclusion of participants?	Unclear
	5. Did the case series have complete inclusion of participants?	Yes
	6. Was there clear reporting of the demographics of the participants in the study?	Partial
	7. Was there clear reporting of clinical information of the participants?	Yes
	8. Were the outcomes or follow up results of cases clearly reported?	Yes
	9. Was there clear reporting of the presenting site(s)/clinic(s) demographic information?	Yes
	10. Was statistical analysis appropriate?	Yes

Criteria: Lists the individual items from the JBI Critical Appraisal Checklist for Case Series; Addressed: Indicates whether each criterion was met based on the assessment of the study; Yes: The criterion was clearly met; Unclear: The information provided does not clearly indicate whether the criterion was met or not; Partial: Some aspects of the criterion were met, but not all.

**Supplementary Table 8.** The key clinical application of IRT in different types of arthritis.

**Author/Year**	**Clinical application**
Alfieri et al., 2020^25^	Detect subtle knee temperature increases in unilateral knee OA.
Arfaoui et al., 2012^26^	IRT is a reliable, non-invasive tool for detecting increased skin temperature patterns in knee osteoarthritis, correlating with patient-reported pain levels. It may enable early detection of knee osteoarthritis before radiographic changes appear.
Balay-Dustrudeet al., 2023^65^	Detecting knee inflammation in children with active arthritis.
Brito et al., 2020^52^	Thermography may be useful for monitoring effects of exercise in osteoarthritis patients.
Bhowmik et al., 2016^27^	IRT aids in quantifying abnormal inflammation and pain associated with arthritis by assessing heat emission from the skin surface. This technique proves valuable in distinguishing between OA and RA by identifying distinct thermal patterns indicative of each condition.
De et al., 2023^28^	IRT was used clinically to evaluate inflammation in OA knees by assessing skin temperature and characterising joint response to exercise. It identified different OA patterns to guide personalised treatment and studied specific joint subareas to better understand inflammatory components.
Denoble et al., 2010^49^	Potential applications in assessing joint conditions, particularly in identifying associations with disease severity
Devereaux et al., 1985^60^	The summated HDI from infrared thermography was identified as a suitable, objective, reproducible, accurate, and non-invasive method response to therapy in patients with RA.
Frize et al., 2011^32^	Detection of presence of RA.
Gatt et al., 2019^33^	Thermal imaging was considered to have the potential to become an adjunct assessment method for disease activity in patients with RA.
Gatt et al., 2020^34^	Thermal imaging could be used for clinical and self-monitoring to detect temperature changes in the feet of patients with RA.
Gizińska et al., 2021^35^	Thermal imaging can support the diagnosis and assessment of treatment effectiveness for diseases like RA. By analysing the temperature distribution of the feet, clinicians can diagnose RA and monitor disease progression.
Goldie et al., 1974^63^	Useful for assessing the efficacy of anti-inflammatory drugs.
Hegedus et al., 2009^54^	Thermography is used to observe microcirculatory changes during treatment. Increased metabolism and a richer blood supply to tissues result in greater infrared emission.
Jones et al., 2018^61^	No significant clinical application was found.
Lasanen et al., 2015^42^	Thermal imaging may have potential for detecting ankle joint inflammation in children with juvenile idiopathic arthritis or autoimmune diseases like lupus.
Lerkvaleekul et al., 2017^40^	IRT shows promise for assessing wrist arthritis in JIA patients. Parameters such as Tmean and Tmax are effective for detecting moderate to severe arthritis, though they may not be as useful for identifying mild arthritis.
Lindberg et al., 2021^48^	The thermographic method demonstrated its utility in detecting Raynaud’s Phenomenon and could effectively replace the FSP test as a diagnostic tool.
Nwaizu et al., 2020^41^	Assist in examining juvenile idiopathic arthritis by quantifying temperature changes associated with joint inflammation.
Morales-Ivorra et al., 2022^37^	Thermography of the hands, analysed with machine learning, offers a non-invasive, instant, automated, and operator-independent method for assessing joint inflammation. ThermoJIS could identify patients with persistent subclinical joint inflammation, indicating a higher risk of flares and structural damage progression.
De Marziani et al., 2023^53^	IRT may enable personalised treatment by identifying OA patterns linked to demographic and clinical characteristics. It offers an objective measure of knee status, correlating with clinical scores, and could help detect neuropathic pain in OA patients. Additionally, thermography holds potential for non-invasive monitoring of treatment response and for repeated assessments.
Pauk et al., 2019^55^	IRT, coupled with a cold provocation test, shows promise as a diagnostic tool for detecting inflammation in RA patients. Analysing temperature profiles across the fingers and assessing Tmax-Tmin changes following cold stress and rewarming can enhance diagnostic precision, distinguishing RA patients from healthy individuals.
Pauk J et al., 2019^56^	IRT has shown potential in distinguishing disease activity levels in RA patients, offering insights into inflammation and vascular irregularities linked to the condition. Variations in thermal responses, including changes in mean temperature during rewarming, the area under the heating curve, and correlations with clinical parameters, indicate that thermography could complement traditional diagnostic approaches for RA.
Snekhalatha et al., 2015^36^	IRT can serve as a diagnosis tool in RA.
Tan et al., 2020^57^	IRT shows promise as a valuable tool for assessing joint inflammation in RA. The strong correlation observed between thermographic parameters and ultrasound-detected inflammation indicates its potential utility in detecting and monitoring synovitis effectively.
Tan et al., 2021^58^	Potential use of combined thermal and ultrasound imaging to assess joint inflammation and disease activity in rheumatoid arthritis.
Tan et al., 2024^59^	Thermography displayed potential for evaluating joint inflammation at the elbow in patients with RA.
Umapathy et al; 2020^38^	Thermography is proposed as a potential complementary diagnostic tool for the early detection of rheumatoid arthritis in the knee region. A CAD model using thermal imaging could serve as a pre-screening tool to diagnose RA in the knee area.
Varjú et al., 2004^50^	Thermography may offer a non-invasive way to evaluate the presence and severity of osteoarthritis and could potentially be used to monitor the effectiveness of OA treatments.
Vasdev et al., 2023^39^	Thermal imaging offers a simple, non-invasive method to detect joint inflammation and has the potential to become a reliable, cost-effective alternative to ultrasound or MRI for assessing disease activity in inflammatory arthritis.
Vinson et al., 2020^62^	While the clinical application of thermography did not conclusively demonstrate the ability to assess RA activity in small joints, the results were encouraging, showing notable temperature variations in inflammatory joints.
Zhang et al., 202^35^	It can detect physiological and functional changes before structural changes occur, making it valuable for pre-treatment evaluation.
Zhao et al., 2022^64^	Infrared thermal imaging shows promise for clinical use in arthritis detection.

ROI: Regions of interest; IRT: Infrared thermography; OA: Osteoarthritis; RA: Rheumatoid arthritis; ICCs: Intraclass correlation coefficients; MCP: Metacarpophalangeal; T mean: Mean temperature; Tmax: maximum temperature; HDI; Heat Distribution Index; JIA: juvenile idiopathic arthritis; FSP: Finger systolic pressure; ThermoJIS: Thermographic Joint Inflammation Score; AUROC: Area under the receiver operating characteristic curve; DA: Disease activity; ΔTR: Total change in mean temperature of the ROI due to rewarming; SR: the area under the cooling curve; TPD: Total power doppler; TGS: Total grey-scale; MIN: Minimum; MAX: Maximum; AVG: Average; CAD: Computer-aided diagnostic; rOA: Radiographic osteoarthritis; KL: Kellgren-Lawrence; ΔT: Temperature difference; TAWiC: Temperature after within-limb calibration.

**Supplementary Table 9.** The key clinical application of IRT in other rheumatic diseases.

**Author/Year**	**Clinical application**
Abreau et al., 2016^24^	Evaluate ocular surface temperature and the thermal profile of the eyelids and periorbital regions in dry eye patients with different aetiologies.
Agazzi et al., 2018^43^	A helpful non-invasive tool for detecting disease activity JLS lesions. It can reliably monitor changes in lesion activity/inflammation.
Bali et al., 2011^70^	The study used thermography to objectively measure changes in digital temperature as an outcome measure for evaluating the effects of iloprost treatment in patients with RP and Ssc.
Casas-Barragán et al., 2021^29^	IRT demonstrated utility in diagnosing conditions, assessing disease severity, evaluating sleep disturbances, and measuring fatigue and pain in FMS patients.
Coleiro et al., 2001^73^	IRT appears to be a valuable objective tool for assessing vascular reactivity and treatment responses in Raynaud's phenomenon.
Dziadzio et al., 1999^74^	The study used thermography to estimate skin temperature and assess vascular response to cold challenge in patients with Raynaud's phenomenon.
Herrick et al., 2014^44^	Thermography can be used as a non-invasive imaging technique to evaluate microvascular function in patients with systemic sclerosis
Kümpel et al., 2023^31^	IRT did not demonstrate sufficient diagnostic accuracy to be recommended as a viable auxiliary method for diagnosing fibromyalgia
Martini et al., 2002^64^	Thermography is effective in detecting active inflammation and increased blood flow in localised scleroderma lesions, monitoring treatment response by assessing changes in lesion warmth over time, and predicting disease progression or arrest through serial examinations.
Martini et al., 2019^45^	Identifying patients with peripheral microvascular disturbances (PRP, SRP, and AC) from healthy controls, and distinguishing between PRP, SRP, and AC based on rewarming patterns and DDD analysis.
Miziołek et al., 2021^65^	IRT may be useful in evaluating microvasculopathy in SSc. IRT can help observe progression from early to late nailfold videocapillaroscopy patterns and appears promising for predicting digital ulcer development.
Miziołek et al., 2023^66^	Potential use in differentiating dcSSc vs lcSSc.
Schlager et al., 2010^67^	IRT is a valuable non-invasive method for assessing skin perfusion, and vasoreactivity in RP.
Schuhfried et al., 2000^46^	IRT can be useful in the diagnosis and evaluation of RP, particularly secondary RP.
Sempere-Rubio et al., 2021^30^	There was no significant reduction or difference in skin temperature at rest for women with fibromyalgia compared to healthy women.
Sternbersky et al., 2021^47^	IRT serves as an effective screening tool for RP. It provides guidance on selecting fingers for focused capillaroscopy examinations and holds potential for assessing treatment efficacy in subsequent studies.
Wilkinson et al., 2018^72^	Thermography is recommended as a secondary outcome measure in clinical trials for Raynaud's phenomenon associated with systemic sclerosis.
Zulian et al., 2011^71^	Thermography was used as an objective measure to evaluate treatment response and disease activity, with a decrease in lesion temperature of ≥10% from baseline considered a criterion for treatment response.

OST: Ocular surface temperature; JLS: juvenile localised scleroderma; IRT: Infrared thermography; mLoSSI: modified Localised Scleroderma Skin Severity Index; FMS: Fibromyalgia Syndrome; MCP: Metacarpophalangeal; RP: Raynaud's phenomenon; PRP: Primary Raynaud's phenomenon; SRP: Secondary Raynaud's phenomenon; AC: Acrocyanosis; DDD: Distal-dorsal difference; Tavg: average temperature; LDPI: laser Doppler perfusion imaging; SSc; Systemic sclerosis; dcSSc: diffuse cutaneous disease; lcSSc: limited cutaneous involvement; TΔ: temperature gradients; ΔTh%: Percentage thermal change from baseline.
